# How well does the minimum data set measure healthcare use? a validation study

**DOI:** 10.1186/s12913-018-3089-7

**Published:** 2018-04-11

**Authors:** Malcolm B. Doupe, Jeff Poss, Peter G. Norton, Allan Garland, Natalia Dik, Shauna Zinnick, Lisa M. Lix

**Affiliations:** 1 Department of Community Health Sciences, Max Rady College of Medicine, Rady Faculty of Health Sciences, University of Manitoba, 408-727 McDermot Avenue, Winnipeg, MB R3E 3P5 Canada; 2 Manitoba Centre for Health Policy, University of Manitoba, 408-727 McDermot Avenue, Winnipeg, MB R3E 3P5 Canada; 3 School of Public Health and Health Systems, University of Waterloo, 200 University Avenue W, Waterloo, ON N2L 3G1 Canada; 4 University of Calgary, 2500 University Dr NW, Calgary, AB T2N 1N4 Canada; 5 Faculty of Health Sciences, University of Manitoba, 820 Sherbrook St, Winnipeg, MB R3A 1R9 Canada; 6 George & Fay Yee Centre for Healthcare Innovation, University of Manitoba, 4th floor, 753 McDermot Avenue, Winnipeg, MB R3E 0T6 Canada

**Keywords:** Nursing homes, Healthcare use, MDS records, Validation

## Abstract

**Background:**

To improve care, planners require accurate information about nursing home (NH) residents and their healthcare use. We evaluated how accurately measures of resident user status and healthcare use were captured in the Minimum Data Set (MDS) versus administrative data.

**Methods:**

This retrospective observational cohort study was conducted on all NH residents (*N* = 8832) from Winnipeg, Manitoba, Canada, between April 1, 2011 and March 31, 2013. Six study measures exist. NH user status (newly admitted NH residents, those who transferred from one NH to another, and those who died) was measured using both MDS and administrative data. Rates of in-patient hospitalizations, emergency department (ED) visits without subsequent hospitalization, and physician examinations were also measured in each data source. We calculated the sensitivity, specificity, positive and negative predictive values (PPV, NPV), and overall agreement (kappa, *κ*) of each measure as captured by MDS using administrative data as the reference source. Also for each measure, logistic regression tested if the level of disagreement between data systems was associated with resident age and sex plus NH owner-operator status.

**Results:**

MDS accurately identified newly admitted residents (*κ* = 0.97), those who transferred between NHs (*κ* = 0.90), and those who died (*κ* = 0.95). Measures of healthcare use were captured less accurately by MDS, with high levels of both under-reporting and false positives (e.g., for in-patient hospitalizations sensitivity = 0.58, PPV = 0.45), and moderate overall agreement levels (e.g., *κ* = 0.39 for ED visits). Disagreement was sometimes greater for younger males, and for residents living in for-profit NHs.

**Conclusions:**

MDS can be used as a stand-alone tool to accurately capture basic measures of NH use (admission, transfer, and death), and by proxy NH length of stay. As compared to administrative data, MDS does not accurately capture NH resident healthcare use. Research investigating these and other healthcare transitions by NH residents requires a combination of the MDS and administrative data systems.

**Electronic supplementary material:**

The online version of this article (10.1186/s12913-018-3089-7) contains supplementary material, which is available to authorized users.

## Background

Nursing homes (NHs) have become increasingly complex care environments and are in growing demand. The number of 75+ year olds living in both Canada [1] and the United States [2] is expected to double by 2030, and in any given year 1.4 million U.S. citizens [3] and 225,000 Canadians [4] reside in a NH. NH use patterns have also changed dramatically in recent years, with many residents now admitted later in life and with more complex needs [5–7]. Presently, about half of all newly admitted NH residents require weight-bearing help to complete activities of daily living [8], and many are afflicted with co-morbid diseases plus a range of cognitive, behavior, and continence challenges [7, 9, 10]. Rates of healthcare use (e.g., emergency department [ED] visits, hospitalizations) by NH residents have also increased considerably [11, 12], and better management of these transitions has become a major reform focus [13, 14].

Continued research into these and related areas requires high quality data systems. While some authors report challenges using administrative data to measure patient health status and adverse events (e.g., hospital acquired pressure ulcers) [15], several have shown that these records accurately capture many types of healthcare use without recall bias or loss to follow-up [16–19]. In some instances, however, the breadth of administrative records available and access to them for research varies by geography. Conversely, standardized comprehensive assessments using the interRAI family of instruments is mandated widely across North America [20, 21]. Amongst these, the Resident Assessment Instrument 2.0 (RAI 2.0) exists for NH residents in most Canadian provinces and is comprised of assessment forms (called the Minimum Data Set; MDS), a standard operating manual (provides definitions and assessment guidelines), and Clinical Assessment Protocols (CAPs, used to help develop resident care plans) [20]. MDS assessments richly define NH residents by (for example) their functional dependence, cognitive impairment, and use of healthcare services including prescription drug use. Validation has been conducted on various MDS scales that measure cognitive performance [22], pain [23], and depression [24]. Authors have also examined the accuracy of MDS quality care metrics [25], plus specific MDS items that capture resident prescription drug use [26, 27] and chronic disease [28, 29]. With some exceptions [30], the accuracy with which MDS captures resident healthcare use is understudied. The present research helps to fill this gap by linking MDS to administrative data for a population of NH residents from Winnipeg, Manitoba. The objective of our study is to assess the accuracy of MDS for capturing: i) NH resident admission, transfers between NHs, and death; and ii) hospital use, ED transfers, and ambulatory care physician visits. These study results define the extent to which MDS data can be used as a stand-alone tool for providing key NH use and healthcare transition information pertinent to guiding NH care reform.

## Methods

### Research environment and study cohort

Manitoba is one of 10 Canadian provinces and has a population of 1.3 million people dispersed across five geographically diverse regions responsible for delivering healthcare. Four of these regions are rural or remote and the Winnipeg Health Region (WHR, the city of Winnipeg) is the only large metropolitan area (population 725,000). About two-thirds of Manitoba’s total NH beds (*N* = 9586) are located in Winnipeg (*N* = 5636 beds across 38 facilities) [31]. This study was conducted on the population of Manitobans who resided in a Winnipeg NH for a least 1 day between April 1, 2011 and March 31, 2013.

### Data sources

The data sources used to conduct this research are described in Table 1. The national standard for reporting MDS data has been established by the Canadian Institute of Health Information, CIHI; https://www.cihi.ca/en). All NH residents are required to have an MDS admission (provides an admission date and an ‘admission from’ location) and discharge (provides the reason and date of discharge) record. A full-length assessment is also required for each resident at NH admission and annually thereafter, interspersed by abbreviated quarterly assessments. Each full assessment contains responses to about 400 standardized items that profile residents by various clinical (e.g., cognitive performance) and healthcare use (e.g., emergency department use) domains. Each assessment is completed by a trained assessor (usually a nurse) using all available information including clinical charts and observations made by the family, staff, physicians, and volunteers.

**Table 1:** Data Sources and Methods Used to Create Study Measures

Study Measures	Administrative Health Care Use Records		InterRAI (MDS) Assessments	
NURSING HOME (NH) USER STATUS	Source	Method	Source	Method
i) Residents who were Newly Admitted During the Study Period (versus not)	NH Utilization File	This file was reviewed retrospectively until 1984, to identify residents admitted during the study period who had not been previously admitted to a NH.	Admission Record	The first admission record was selected for each person. People were identified as a newly admitted NH resident if item: i) AB2a (denotes where the person was admitted from) ≠ 4 (denotes a 24-h nursing care facility); or ii) AB2A = 4 & AB2b (the facility # the person was admitted from) ≠ a Manitoba-based NH ID.
ii) Residents Newly Admitted into a NH Directly from Hospital vs Another Location (*calculated on people defined as newly admitted in both data systems)*	Hospital Discharge Abstract Database (DAD) linked to the NH Utilization File	DAD hospital discharge dates were aligned with NH admission dates. People transferring into NH directly from hospital had ≤1 day between these dates.	Admission Record	People were defined as transferring directly from hospital if item AB2a on this record was coded as any of 1 (inpatient acute care), 2 (inpatient rehab), 5 (inpatient psychiatry) or 7 (inpatient specialized rehab).
iii) Residents with 1+ NH Transfer During the Study Period (versus not)	NH Utilization File	This file contains a NH ID for each resident. Residents who transferred NHs had multiple NH IDs in this file, and the date of the ID change was also captured.	Admission Record	Residents were defined as having transferred NHs if: i) item AB2a = 4 (resident arrived from another NH); or ii) AB2b (facility # admitted from) contained a Manitoba NH ID which did ≠ the present facility ID.
iv) Residents who Died (versus who did not) During the Study Period	Registry File	This file contains a unique identifier for every Manitoban. The Insurance Cancellation Code in this file was used to define the date of each person’s death.	Discharge Record	The last discharge record was selected for each person. People were identified as dying if item i) R3a = 11 (denotes people who were deceased); or ii) AA8 = 6 (discharged and not likely to return) and R3a (discharge locations) was 1 (inpatient acute care), 2 (inpatient rehab), 5 (inpatient psychiatry) or 7 (inpatient specialized rehab).
HEALTH CARE USE				
i) In-patient Hospitalization (yes vs no, and frequency of visits for people with 1+ hospitalization in each data system).	Hospital Discharge Abstract Database (DAD)	DAD was used to count the number of times people were hospitalized with a length of stay > 1 day, overall and within each MDS episode.	MDS full assessments	Item P5 records the number of times each person was admitted to a hospital in the previous 90 days. This 90-day period was defined as the MDS episode.
ii) Emergency Department Visits not ending in Hospitalization (yes vs no, and frequency of visits for people with 1+ ED visit in each data system).	The Emergency Department Information System (EDIS)	This file was used to count the number of times each person visited an ED, overall and within each MDS episode. Visits ending in hospitalization were excluded using the EDIS disposition code.	MDS full assessments	Item P6 records the number of times each person visited the ED without being hospitalization in the previous 90 days. This 90-day period was defined as the MDS episode.
iii) Number of Days the Resident was Examined by a Physician (0 versus 1+ days; and frequency of days for people with 1+ examination in each data system).	The Medical Claims File*	This file was used to count the number of days people who had an ambulatory care physician visit (e.g., where the patient was cared for in the NH), overall and within each MDS episode. Ambulatory care visits are defined in a table footnote.	MDS full and quarterly assessments	Item P7 records the number of days each person was examined by a physician (or authorized professional such as a nurse practitioner) in the previous 14 days. This 14-day period was defined as the MDS episode.

*Prefix ‘7’ tariff codes denote ambulatory care physician visits in medical claims. Tariff code 8511 (‘general scheduled visit for chronic care’) accounts for 77.7% of all ambulatory care physician visits measured in the study period, code 8513 (‘regular visit for patients aged 70 years and older’) accounts for 9.1% of all such visits, and tariff code 8500 (‘complete physical exam for patients aged 70 years and older’) accounts for 3.4% of all ambulatory care physician visits measured in the study period

Administrative data have been available in Winnipeg since 1984, although some data sources originate before this date. The Registry File contains a unique identifier for every Manitoban including their birth and death date, and is used for linkage to all other files. The NH Use File contains the dates of admission and discharge for every NH resident in Manitoba, while the Hospital Discharge Abstract Database (DAD) provides the dates of hospital admission and discharge plus reasons for hospitalization. The Emergency Department Information System (EDIS) provides the dates of ED visits plus details about the care provided during each visit, and the Medical Claims File provides the dates and types of physician visits as well as the primary reason for the visit.

The standards for capturing data in most administrative files are managed centrally by the Government of Manitoba. Certified health information management professionals are responsible for creating all DAD abstracts, and the Registry File receives weekly birth and death updates from Vital Statistics. Re-abstracting studies completed by CIHI conclude that DAD data are of particular high quality in Manitoba, especially for capturing patient diagnoses and procedures [32]. Previous research has demonstrated that the Medical Claims File provides diagnostic and procedural data that are highly comparable to other information sources [16]. Researchers have also shown that 97.5% of ED patients recorded as being hospitalized in EDIS were found in the DAD on the same day [33].

Both MDS and administrative data are housed at the Manitoba Centre for Health Policy, Max Rady College of Medicine, University of Manitoba. Unique, person-level and time-stamped identifiers exist in each of these files, enabling researchers to link them when studying system-level patterns of healthcare use. Further details about general data linkage processes are available elsewhere [17, 18].

### Measures of NH user status and healthcare use

The study measures are described in Table 1. Measures of NH user status include being newly (i.e., first time) admitted into the NH system, having transferred between NH facilities one or more times, and having died during the study period. For residents defined as newly admitted in both data systems, we also measured whether they were admitted directly from hospital or another location.

Measures of healthcare use include in-patient hospitalizations, ED visits not resulting in hospitalization, and ambulatory care physician visits. These measures were assessed during specific times (episodes) as predicated by the MDS assessment (called ‘look-back’ episodes in this manuscript). During each full MDS assessment, assessors are asked to record the number of times the resident was admitted to a hospital in the last 90 days, and to curtail this look-back episode if a previous full assessment occurred during this time. Based on these criteria, the equivalent date-stamped episodes were created using DAD (with 2 days of overlap to account for data recording errors), and the number of hospital discharges during each episode was compared between data systems. Also, some of these look-back episodes include days preceding a resident’s NH admission date. Comparisons of hospital use across data systems were therefore made separately on assessments completed within the first 90 days of NH admission (i.e., where at least some of the ‘look-back’ episode preceded the resident’s stay in a given NH), and all others.

This same strategy was used to identify ED visits not resulting in hospitalization (Table 1). Counts of these visits are recorded during full MDS assessments only. Assessors are asked to record the number of these visits in the last 90 days, curtailed if previous full assessments occurred during this time. The equivalent time-stamped episodes (plus or minus 2 days to account for error) were created using EDIS. Counts of ED visits were compared across data systems during each episode, stratified by assessments completed within or following each resident’s first 90 days of NH stay.

Physician visits are captured in MDS during both full and quarterly assessments. Assessors recorded how often residents were examined by a physician (or a related care provider such as a nurse practitioner or dentist with additional training) in the past 2 weeks. Tariff codes from the Medical Claims file were used to identify a subset of physician visits (i.e., ambulatory care or examinations in the NH) that occurred during each MDS look-back episode, and a list of the major tariff codes used to identify ambulatory care physician visits is provided in Table 1. Comparisons across data systems identify the number of residents who were examined during zero versus one or more days during each look-back episode.

### Statistical analysis

The study cohort was first described by the number of different MDS records (i.e., assessment and discharge records, full and quarterly assessments) that existed; and then by resident age (< 65, 65–74, 75–84, 85+ years), sex, and whether people lived in a NH that was not-for-profit, for profit, or a combination of these ownership types during the study period. Measures of NH user status and healthcare use were then compared between the MDS and administrative data systems, using the latter as the reference data source. The unit of analysis for NH user status measures is the person, while the unit of analysis for measures of health care use is the MDS look-back episode.

The accuracy of each study measure was calculated. Sensitivity was defined as proportion of true cases correctly identified by MDS, while specificity was defined as the proportion of true negatives correctly identified by MDS. Positive predictive value (PPV) was defined as the proportion of all cases defined by MDS that were truly positive, while negative predictive value (NPV) was defined as the proportion of all non-cases in MDS that were truly negative. Cohen’s kappa coefficient (*κ*) [34] was used to measure overall agreement between these data sources. As recommended by Cohen, kappa values can be categorized as: i) < 0.20 (poor); ii) 0.20 to 0.39 (fair); iii) 0.40 to 0.59 (moderate); iv) 0.60 to 0.79 (good); and v) 0.80 to 1.0 (very good).

Multiple logistic regression was conducted on each study measure to determine if the level of disagreement between data systems varied by resident age and sex, plus NH owner-operator type. For each model, residents who were similarly identified by each data system (e.g., newly admitted by both MDS and administrative records) were included in the agreement category (reference group), while those who were dis-similarly identified were included in the disagreement category. Model estimates are presented as adjusted odds ratios (AORs) with 95% confidence limits (95% CLs). Given the hierarchical nature of these data, a random intercept was included for each NH to account for the clustering of residents within facilities. Using the strategies outlined by Ene (2014), the intraclass correlation coefficient (ICC) was computed to determine what proportion of the total variation in study outcomes was accounted for by NH facilities [35]. Concordance (C) statistics were calculated to measure how well each model discriminated across agreement categories. This value ranges from 0.5 (no discrimination) to 1.0 (perfect discrimination). All analyses were conducted using SAS version 9.4.

## Results

The study cohort included 8832 NH residents with 52,818 MDS records (Table 2). A small number of residents (*N* = 137) had no MDS data, while just under half had at least one admission (41.4%) and discharge (46.2%) record. Full MDS assessments were completed on 90.5% of residents, and 60.5% of residents had one or more quarterly assessments.

**Table 2:** Counts of MDS Records and Characteristics of the Study Cohort

	People (*N* = 8832)	Assessments (*N* = 52,818)
A) MDS RECORDS^a^		
No record	137 (1.6)	0 (0)
Admission record	3657 (41.4)	4463 (8.4)
Full assessment record	7977 (90.5)	24,905 (47.2)
Quarterly assessment record	5346 (60.5)	19,098 (36.2)
Discharge record	4083 (46.2)	4352 (8.2)
B) STUDY COHORT		
Resident Age		
< 65	369 (4.2)	2444 (4.6)
65–74	771 (8.7)	4800 (9.1)
75–84	2742 (31.0)	16,502 (31.2)
85+	4950 (56.1)	29,072 (55.1)
Resident Sex		
Male	2693 (30.5)	15,619 (29.6)
Female	6139 (69.5)	37,199 (70.4)
Nursing Home Owner Operator Type		
For Profit	3252 (36.8)	17,820 (33.7)
Not-for-profit	4973 (56.3)	30,093 (60.0)
Both^b^	607 (6.9)	4905 (9.3)

^a^People had multiple MDS records. Counts of people by assessment type therefore exceed the total cohort size

^b^Represents people who transferred between a for profit & not-for-profit nursing home during the study period

The distribution of MDS records was similar by resident age and sex (Table 2); 56.1% of the study cohort was 85+ years old and 55.1% of MDS records were completed on these residents. Similarly, 4.2% of the study cohort was younger than 65 years old and 4.6% of MDS records were completed on these people. Also, 69.5% of the study cohort was female and 70.4% of MDS records were completed for females. Similar results exist for NH owner-operator status (e.g., 36.8% of the study cohort resided in a for-profit NH during the study period and 33.7% of all MDS assessments were completed on residents who resided in these NHs).

**Fig. 1:**
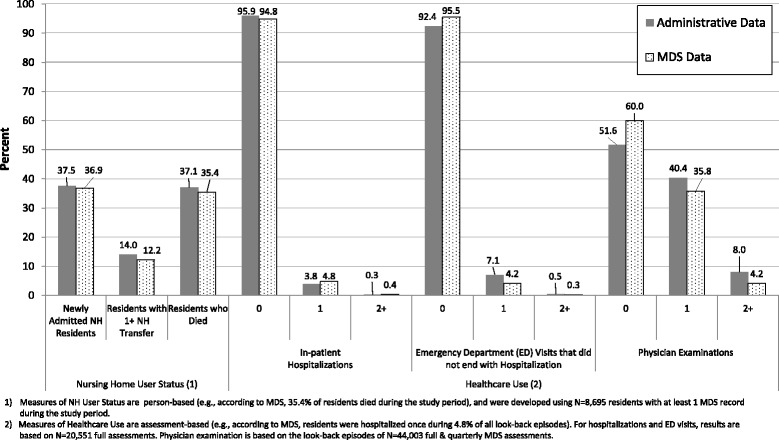
Nursing Home User Status and Episodes of Healthcare Use, by Data Source

Administrative data revealed that 37.5% of NH residents were newly admitted during the study period, while MDS revealed that 36.9% of residents were newly admitted during this time (Fig. 1; contingency tables showing denominators are provided in Additional file 1). Similarly, 37.1 and 35.4% of residents were reported to have died during the study period, using administrative and MDS data, respectively. Administrative data revealed that 14.0% of residents transferred between NHs at least once during the study period, versus 12.2% of residents as defined using MDS.

Counts of healthcare use between data systems are also reported (Fig. 1). Residents were reported to have not been hospitalized during 95.9 and 94.8% of look-back episodes, using the administrative and MDS data, respectively. Similarly, administrative data revealed that residents did not have ED visits ending in hospitalization during 92.4% of look-back episodes, versus 95.5% of look-back episodes reported by MDS. Residents were not examined by a physician during 51.6 and 60.0% of look-back episodes, as reported using administrative and MDS data, respectively. MDS also underreported the frequency of physician examinations during these times (i.e., residents were examined by physician on multiple days during 8.0 and 4.2% of all look-back periods, using administrative and MDS data, respectively).

Comparisons of these study outcomes were examined in more detail (Table 3; Additional file 1). Measures of sensitivity, specificity, PPV, and NPV values were all above 0.86 for measures of NH user status, and in most instances these values were greater than 0.96. Kappa (*κ*) values ranged from 0.90 (NH transfers) to 0.97 (newly admitted residents), indicating very high levels of overall agreement. For residents identified as newly admitted in both systems, MDS also accurately defined residents who transferred into NH from hospital versus from other locations (e.g., *κ*=0.74). The median (10th, 90th percentile) difference in time between NH admission dates in these systems was 0 (0, 7) days (data not shown). Similarly, for residents who were reported as dying in both systems, the median (10th, 90th percentile) difference in time between death dates was 0 (0, 0) days (data not shown).

**Table 3:** Levels of Agreement Between MDS and Administrative Health Care Use Records; Value (95% Confidence Limits)

	Count	Sensitivity	Specificity	Positive Predicted Value	Negative Predicted Value	Kappa
Nursing Home User Status						
i) Newly Admitted NH Residents	8695	0.97 (0.96; 0.98)	0.99 (0.99; 1.00)	0.99 (0.98; 0.99)	0.98 (0.98; 0.99)	0.97 (0.96; 0.97)
ii) Residents Admitted from Hospital versus Another Location^a^	3164	0.91 (0.90; 0.92)	0.82 (0.80; 0.84)	0.91 (0.90; 0.92)	0.83 (0.81; 0.85)	0.74 (0.71; 0.76)
ii) Residents with 1+ NH Transfer	8695	0.86 (0.84; 0.88)	0.99 (0.99; 0.99)	0.98 (0.97; 0.99)	0.98 (0.97; 0.98)	0.90 (0.89; 0.92)
iii) Residents who Died	8695	0.94 (0.93; 0.95)	0.99 (0.99; 1.00)	0.99 (0.99; 0.99)	0.97 (0.96; 0.97)	0.95 (0.94; 0.95)
Health Care Use						
i) Hospital Inpatient Visits^b^	20,551	0.58 (0.55; 0.61)	0.97 (0.97; 0.97)	0.45 (0.42; 0.48)	0.98 (0.98; 0.98)	0.49 (0.46; 0.51)
Frequency of visits^c^	484	0.60 (0.46; 0.72)	0.90 (0.86; 0.92)	0.41 (0.30; 0.52)	0.95 (0.92; 0.97)	0.41 (0.29; 0.52)
ii) ED Visits not ending in Hospitalization^b^	20,551	0.34 (0.32; 0.36)	0.98 (0.98; 0.98)	0.57 (0.54; 0.60)	0.95 (0.94; 0.95)	0.39 (0.37; 0.42)
Frequency of visits^c^	532	0.34 (0.26; 0.44)	0.92 (0.90; 0.95)	0.54 (0.42; 0.65)	0.85 (0.81; 0.88)	0.31 (0.21; 0.41)
iii) Days of Physician Examinations	44,003	0.52 (0.52; 0.53)	0.72 (0.71; 0.72)	0.63 (0.63; 0.64)	0.62 (0.61; 0.62)	0.24 (0.23; 0.25)
Frequency of days^c^	11,135	0.24 (0.23; 0.26)	0.87 (0.86; 0.88)	0.47 (0.44; 0.49)	0.71 (0.70; 0.72)	0.13 (0.11; 0.15)

^a^Conducted on residents defined as newly admitted in both data systems

^b^Results exclude MDS assessments completed within 90 days of any NH admission (*N* = 4354)

^c^Analyses conducted on residents with one or more health care contacts in each data system

As compared to administrative data, measures of healthcare use in MDS are characterized by high levels of specificity (e.g., 0.97 for hospitalizations) and NPV (e.g., 0.97 for ED visits not ending in hospitalization). However, considerable underreporting and false positives in MDS occurred (Table 3; Additional file 1). MDS correctly identified hospitalizations during only 58% of the look-back episodes (i.e., sensitivity = 0.58) and conversely, often over-reported hospitalizations (PPV = 0.45). Similarly, during periods where agreement between data systems was found, MDS both underestimated (sensitivity = 0.60) and over-reported (PPV = 0.41) how often multiple hospitalizations occurred. This same pattern of results exists for non-hospitalized ED visits (sensitivity = 0.34, PPV = 0.57) and days of physician examinations (sensitivity = 0.51, PPV = 0.71). Kappa values were correspondingly low for all of these measures, ranging from 0.24 for days of physician examinations to 0.49 for in-patient hospitalizations. These results for hospitalizations and ED visits exclude assessments (*N* = 4354) completed within the first 90 days of NH admission. Outcomes on this subset of assessments were marginally worse than those reported in Table 4 (hospital admissions: Sens = .41, Spec = .96, PPV = .59, NPV = .93, *κ*=.43; ED visits without hospitalization: Sens = .29, Spec = .97, PPV = .49, NPV = .93, *κ*=.32).

**Table 4:** Adjusted Odds Ratios (95% Confidence Limits) Comparing the Disagreement between MDS and Administrative Data

	Newly Admitted Residents	Residents Admitted from Hospital versus Another Location	Residents with 1+ NH Transfer	Residents who Died	Hospital Inpatient Visits	ED Visits not ending in Hospitalization	Days of Physician Examinations
Resident Age (ref = < 65 years old)							
65–74 years old	0.9 (0.4, 2.0)	1.0 (0.5, 2.1)	0.6 (0.3, 1.3)	1.1 (0.5, 2.6)	0.8 (0.6, 1.1)	0.9 (0.7, 1.2)	1.1 (0.9, 1.1)
75–84 years old	0.6 (0.3, 1.3)	1.4 (0.7, 2.6)	0.8 (0.4, 1.4)	0.9 (0.4, 2.0)	0.8 (0.6, 1.1)	1.0 (0.8, 1.4)	1.1 (0.9, 1.2)
85+ years old	0.3 (0.2, 0.7)†	1.2 (0.6, 2.2)	0.3 (0.2, 0.6)‡	1.0 (0.5, 2.1)	0.7 (0.5, 1.0)*	1.0 (0.8, 1.3)	1.0 (0.9, 1.2)
Resident Sex (ref = Female)							
Male	0.8 (0.5, 1.1)	1.0 (0.7, 1.2)	1.6 (1.1, 2.1)†	1.5 (1.1, 2.0)†	1.1 (0.9, 1.3)	1.0 (0.9, 1.1)	1.0 (0.9, 1.0)
Nursing Home Owner Operator Type (ref = Not-for-profit)							
For Profit	1.6 (0.9, 2.8)	1.2 (0.6, 2.2)	0.7 (0.4, 1.1)	0.5 (0.3, 0.7)‡	1.3 (1.0, 1.7)*	1.2 (0.9, 1.6)	1.0 (0.9, 1.2)
Both^	5.1 (2.8, 9.0)‡	1.5 (0.8, 2.8)	11.7 (7.9, 17.4)†	0.9 (0.6, 1.6)	1.4 (1.0, 1.8)*	1.2 (0.9, 1.5)	1.1 (0.9, 1.2)
Model Parameters							
Intraclass Correlation (ICC)	0.22	0.22	0.14	0.11	0.03	0.05	0.03
C Statistic	0.64	0.58	0.77	0.60	0.57	0.54	0.52

*p < .05; †*p* < .01; ‡*p* < .001

^Represents people who transferred between a for profit & not-for-profit nursing home during the study period

AORs (Table 4) show that the odds of disagreement between data systems was consistent for some but not all residents. Disagreement was significantly lower for residents 85+ years versus < 65 years when defining newly admitted residents (AOR = 0.3; 95% CL = 0.2, 0.7), residents with one or more NH transfers (AOR = 0.3; 95% CL = 0.2, 0.6), and hospitalizations (AOR = 0.7; 95% CL = 0.5, 1.0). The odds of disagreement was significantly higher for males when measuring NH transfers (AOR = 1.6; 95% CL = 1.1, 2.1) and resident death (AOR = 1.5; 95% CL = 1.1, 2.0). In some instances (new admission, NH transfers, in-patient hospitalization), disagreement between data systems was significantly higher for residents who resided in a for-profit versus not-for-profit NH at some time during the study period. ICC values ranged from 0.03 (days of physician examination and hospital inpatient visits) to 0.22 (newly admitted residents and their transfer from location). C-statistic values were 0.60 and lower for all outcomes except when defining newly admitted residents and those with one or more NH transfer, indicating that model covariates did not effectively discriminate between agreement categories.

## Discussion

This study investigates how accurately key measures of NH user status and healthcare use were captured in the InterRAI Minimum Data Set (MDS) assessment instrument. Our results show that MDS accurately defined NH residents by their admission and death dates (and therefore length of stay), and also accurately identified newly admitted NH residents who transitioned from hospital and who transferred between NH facilities. However, as compared to administrative data, MDS both undercounted the true rate of healthcare use and provided false positives. Using Cohen’s agreement criteria [34], kappa values for these time specific comparisons were fair when measuring physician visits and non-hospitalized ED visits, and moderate when measuring in-patient hospitalizations.

These results are supported by some existing literature. Mor et al. (2011) report that MDS accurately defines resident death (sensitivity = 0.84, PPV = 0.95) as compared to administrative data [30], but show that 20% of hospitalizations reported in MDS were not found in administrative records. Similarly, Lix et al. (2015) show that MDS records accurately defined anti-psychotic and anti-depressant drug users, but observed only moderate agreement with administrative data (k = 0.40) when identifying anti-anxiety or hypnotic drug users [27]. Lix et al. (2014) also report that MDS records (versus administrative data) accurately define NH residents with some (i.e., those with diabetes) but not all types (e.g., congestive heart failure, COPD, stroke) of chronic disease, and often with high rates of both under-reporting and false positives [28]. In their literature review, Hutchinson et al. (2010) report that MDS quality indicators are captured with varying degrees of accuracy, also with under-reporting noted for some metrics and over-reporting noted for others [25]. The present research supports and extends these findings from the existing literature, by showing that MDS can be used to accurately measure NH user status at the population-level, but not necessarily to measure these residents’ healthcare use. As noted by Hirdes et al. (2013), MDS assessments completed using some software systems may be ‘auto-populated’ with responses from the previous assessment, and failure to amend these automatic updates may propagate false positive responses [20].

Accurate data systems are needed to inform NH care innovations. Several authors have investigated the consequences of transferring into NH from hospital [36, 37], have profiled the care needs of newly admitted NH residents [8, 38], and have studied how community-based housing options have impacted NH use [39]. Continued research in these and related areas is feasible using MDS as a stand-alone data source, and have value especially when combining the clinical with user status data available in this tool. Similarly, some authors have defined the extent to which NH bed supply varies by geography [40], and MDS can be used to investigate some of the key consequences (e.g., differences in resident profiles, length of NH stay) of these difference healthcare approaches. Additional research needed to support innovation includes measuring the rate and type of emergency department visits made by NH residents [41], comparing these transition rates to assisted living residents, and defining hospitalizations preceding NH resident death [11, 42]. Continued research in these and related areas requires a combination of the MDS and administrative data systems.

While population-based, the present research was conducted using data from a single urban health region. Just as NH use patterns and resident clinical profiles vary across Canada [7], so too may the results of the present research. In addition to housing MDS records, the CIHI national repository houses administrative data capturing some measures of healthcare use defined in this research. We recommend that the present study be replicated more broadly using CIHI and with jurisdictional comparisons.

Our study results for healthcare use do not account for the nested nature of assessments within residents, and hence the confidence limits for these measures may be too narrow. To partially test this hypothesis, ED use was compared between data sources using one full MDS assessment selected per resident. Results were similar to that shown in the present study (data not shown). Also, while we recognize that the definition of physician visits is broader in MDS (which includes examinations provided by nurse practitioners and dentists with additional training) than in Medical Claims (ambulatory physician only), during the time of this study few nurse practitioners worked in Winnipeg NHs, and dentists do not provide regular NH care in this region. Lastly, our comparisons of healthcare use are confined to the MDS look-back episodes as defined in this research. While MDS collects healthcare use data intermittently, administrative data captures these records continuously. Given these different approaches, MDS considerably underestimates the overall (i.e., daily) measures of healthcare use during the study period; 45.4% versus 17.6% of residents were hospitalized at least once according to the administrative and MDS data, respectively; 51.6% versus 12.3% of residents had one or more ED visits not ending in hospitalization; and 97.6% versus 77.3% of residents were examined by a physician (data not shown).

## Conclusions

This study examines how accurately measures of NH user status and healthcare use are captured in the MDS instrument as compared to administrative data. Our results show that MDS accurately defined key NH user characteristics. However, as compared to administrative data, MDS records both undercounted the true rate of key healthcare use measures and often provided false positives. Future studies measuring these healthcare use patterns require MDS linkage to administrative data.

## Additional file

Additional file 1: Study Outcome Contingency Tables. Data compare the counts of different nursing homes users (e.g., newly admitted residents, those who transferred between nursing homes, people who died) and measures of healthcare use (e.g., hospital inpatient visits, emergency department visits, physician examinations) between the Minimum Data Set system and administrative files. (DOCX 23 kb)

## Abbreviations

AORs: Adjusted odds ratios
CIHI: Canadian Institute of Health Information
CLs: Confidence limits CLs
COPD: Chronic Obstructive Pulmonary Disease
DAD: Hospital Discharge Abstract Database
ED: Emergency department
EDIS: Emergency Department Information System
ICC: Intraclass correlation coefficient
MDS: Minimum Data Set
NH: Nursing home
NPV: Negative predictive values
PPV: Positive predictive values
WHR: Winnipeg health region
