# Supplementary material for: How well does the minimum data set measure healthcare use? a validation study
Source: BMC Health Serv Res. 2018 Apr 11;18:279. doi: 10.1186/s12913-018-3089-7 (PMC5896092; doi:10.1186/s12913-018-3089-7)
Supplement: Supplementary file 1 — Study Outcome Contingency Tables. Data compare the counts of different nursing homes users (e.g., newly admitted residents, those who transferred between nursing homes, people who died) and measures of healthcare use (e.g., hospital inpatient visits, emergency department visits, physician examinations) between the Minimum Data Set system and administrative files. (DOCX 23 kb) [file 12913_2018_3089_MOESM1_ESM.docx]

| **Additional file 1. Study Outcome Contingency Tables** | | | | | | | | | |
| --- | --- | --- | --- | --- | --- | --- | --- | --- | --- |
| 1. **Measures of Nursing Home User Status** | | | | | | | | | |
| **i) Newly Admitted Residents** | | | | | **iii) Residents with 1+ NH Transfer** | | | | |
|  |  | Administrative Data | |  |  |  | Administrative Data | |  |
|  |  | Admit | No Admit | Totals |  |  | Transfer | No Transfer | Totals |
| MDS (admission assessment) | Admit | 3,164 | 33 | 3,197 | MDS (admission assessment) | Transfer | 1,044 | 18 | 1,062 |
|  | No Admit | 91 | 5,407 | 5,498 |  | No  Transfer | 171 | 7,462 | 7,633 |
|  | Totals | 3,255 | 5,440 | 8,695 |  | Totals | 1,215 | 7,480 | 8,695 |
| **ii) Residents Admitted from Hospital versus Another Location*** | | | | | **iv) Residents who Died** | | | | |
|  |  | Administrative Data | |  |  |  | Administrative Data | |  |
|  |  | Hospital | Else | Totals |  |  | Death | No Death | Totals |
| MDS (admission assessment) | Hospital | 1,911 | 191 | 2102 | MDS (discharge assessment) | Death | 3,044 | 31 | 3,075 |
|  | Else | 181 | 881 | 1062 |  | No Death | 185 | 5,435 | 5,620 |
|  | Totals | 2,093 | 1,071 | 3,164 |  | Totals | 3,229 | 5,466 | 8,695 |
| * Analyses conducted on residents defined as newly admitted in both data systems | | | | | | | | | |
| 1. **Measures of Health Care Use** | | | | | | | | | |
| **a) OVERALL USE** | | | | | **b) FREQUENCY OF USE^** | | | | |
| *i) Hospital Inpatient Visits** | | | | | | | | | |
|  |  | Administrative Data | |  |  |  | Administrative Data | |  |
|  |  | 1+ | 0 | Totals |  |  | 2+ | 1 | Totals |
| MDS (Full Assessments Only) | 1+ | 484 | 582 | 1,066 | MDS (Full Assessments Only) | 2+ | 31 | 45 | 76 |
|  | 0 | 349 | 19,136 | 19,485 |  | 1 | 21 | 387 | 408 |
|  | Totals | 833 | 19,718 | 20,551 |  | Totals | 52 | 432 | 484 |
| *ii) Non-hospitalized ED Visits** | | | | | | | | | |
|  |  | Administrative Data | |  |  |  | Administrative Data | |  |
|  |  | 1+ | 0 | Totals |  |  | 2+ | 1 | Totals |
| MDS (Full Assessments Only) | 1+ | 532 | 406 | 938 | MDS (Full Assessments Only) | 2+ | 37 | 32 | 69 |
|  | 0 | 1,028 | 18,585 | 19,613 |  | 1 | 71 | 392 | 463 |
|  | Totals | 1,560 | 18,991 | 20,551 |  | Totals | 108 | 424 | 532 |
| *iii) Days of Physician Examinations* | | | | | | | | | |
|  |  | Administrative Data | |  |  |  | Administrative Data | |  |
|  |  | 1+ | 0 | Totals |  |  | 2+ | 1 | Totals |
| MDS (Full & Quarterly Assessments) | 1+ | 11,135 | 6,453 | 17,588 | MDS (Full & Quarterly Assessments) | 2+ | 856 | 981 | 1,837 |
|  | 0 | 10,158 | 16,257 | 26,415 |  | 1 | 2,665 | 6,633 | 9,298 |
|  | Totals | 21,293 | 22,710 | 44,003 |  | Totals | 3,521 | 7,614 | 11,135 |
| * Results exclude assessments completed within 90 days of any NH admission (N=4,354).  ^ Analyses conducted on residents with one or more health care contacts in each data system. | | | | | | | | | |
